# 2026 focsused update incorporated into the Japanese society of echocardiography 2023 practice guidance for the implementation of stress echocardiography “stress echocardiography using the Valsalva maneuver”

**DOI:** 10.1007/s12574-026-00735-0

**Published:** 2026-06-08

**Authors:** Yutaka Hirano, Hiroyuki Iwano, Takahiro Ohara

**Affiliations:** 1 Kansai International Airport Clinic, Kindai University Faculty of Medicine, Kansai International Airport Clinic, Kindai University Faculty of Medicine , Osaka, Japan; 2 Department of Cardiovascular Medicine, Teine Keijinkai Hospital, Sapporo, Japan; 3 Division of Geriatric and Community Medicine, Tohoku Medical and Pharmaceutical University, Sendai, Japan

**Keywords:** Stress echocardiography, Valsalva maneuver, Patent foramen ovale, Hypertrophic obstructive cardiomyopathy, Left ventricular diastolic function

## Abstract

In 2023, Based on the continued evidence of stress echocardiography, the new practical guideline that describes the safe and effective methodology of stress echocardiography is now created by Guideline Committee of the Japanese Society of Echocardiography and is designed to expand the use of stress echocardiography for valvular heart disease and heart failure with preserved ejection fraction (HFpEF), as well as ischemic heart disease, hypertrophic cardiomyopathy, and pulmonary hypertension. The Valsalva maneuver is essential for evaluating left ventricular diastolic function and diagnosing patent foramen ovale (PFO). Furthermore, in association with the recent insurance coverage for myocardial myosin inhibitors, a new drug for hypertrophic cardiomyopathy, the Valsalva maneuver has become increasingly important in the diagnosis of hypertrophic obstructive cardiomyopathy. Based on the above background, the Guideline Committee of the Japanese Society of Echocardiography prepared the practice guidance for “Stress echocardiography using the Valsalva maneuver” as the focused update incorporated into the “Practice guidance for the implementation of stress echocardiography.”

## Introduction

The Valsalva maneuver has been routinely performed during echocardiography. Although the Valsalva maneuver is categorized as one of the “stress echocardiography”, the Valsalva maneuver during routine echocardiography is not currently reimbursed as a “stress echocardiography” in Japan. Moreover, it has not been included in the “Practice guidance for the implementation of stress echocardiography.” The Valsalva maneuver is essential for evaluating left ventricular diastolic function and diagnosing patent foramen ovale (PFO). Furthermore, in association with the recent insurance coverage for myocardial myosin inhibitors, a new drug for hypertrophic cardiomyopathy, the Valsalva maneuver has become increasingly important in the diagnosis of hypertrophic obstructive cardiomyopathy (HOCM). Based on the above background, the Guideline Committee of the Japanese Society of Echocardiography has developed the practice guidance for “Stress echocardiography using the Valsalva maneuver” as the focused update incorporated into the “Practice guidance for the implementation of stress echocardiography.”

## Principles and methods of the Valsalva maneuver

The Valsalva maneuver, first described by Dr. Antonio Maria Valsalva in 1704, originally involves exhalation against a closed mouth and nose to evacuate pus from the ear [1]. Various physiological changes and responses are produced by the Valsalva maneuver, which has been used to detect pathological conditions.

In clinical practice, applying stress, such as exercise stress, can yield diagnostic information not obtainable at rest. The Valsalva maneuver can be performed with no, or minimal equipment and remains a useful stress method. The maneuver has a long history, and its associated changes in the blood pressure and heart rate have been well described.

### Valsalva maneuver

The Valsalva maneuver increases intrathoracic pressure to at least 30–40 mmHg for a minimum of 10 s by attempting to exhale against a closed glottis after a deep inspiration [1]. Adequate performance of the maneuver requires　sufficient patients’ understanding and cooperation, and several practice attempts are necessary. If patient cooperation or stress is considered insufficient, the examiner may place his/her hand on the patient’s abdomen and instruct them to push the hand back using their abdomen. When evaluating heart murmurs, the Valsalva maneuver should be applied while breathing shallowly because deep inspiration weakens the sound of the maximally inflated lungs [1]. Since the echo window also becomes narrow when the lung is inflated, the same consideration should be applied to echocardiographic evaluation.

Methods to create stress similar to the Valsalva maneuver include the “party balloon method” to inflate a commercially available rubber balloon [2] and the “syringe method” to blow air into a 10-mL syringe and move the inner cylinder [3].

### Normal response to the Valsalva maneuver

#### Phase I

**Fig. 1:**
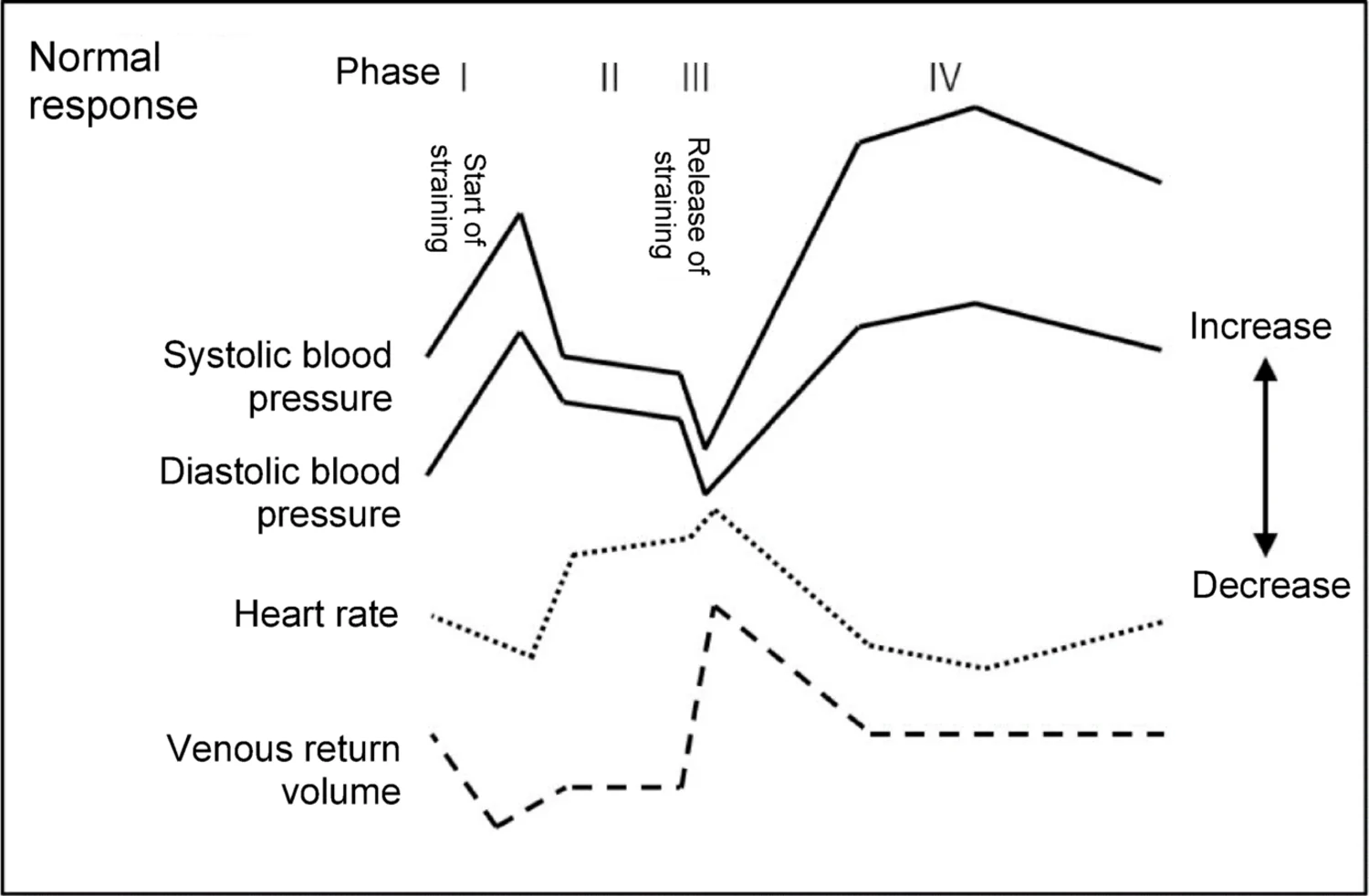
Normal blood pressure/heart rate response to the Valsalva maneuver (prepared based on References 1 and 4–7)

The hemodynamic response to the Valsalva maneuver consist of four phases (Table 1 and Fig. 1) [1]. In phase I immediately after the start of straining, increased intrathoracic pressure elevates aortic pressure for 2 to 3 s [4]. Vagal reflex decreases pulse rate [4]. In the absence of invasive arterial monitoring, these transient blood pressure changes may be difficult to detect at the bedside. During this phase, increased intrathoracic pressure also decreases venous return [5–7].

**Table 1:** Four phases of hemodynamic response to the Valsalva maneuver and normal response [1, 4–7]

Phase	Behavior	Blood pressure	Pulse rate	Venous return	Mechanism
I	Start of straining	Increase	Decrease to no change	Decrease	Increase in intrathoracic pressure
II	Continuous straining	Decrease	Increase	Decrease to maintain	Decrease in venous return → Decrease in pulse pressure and stroke volume
III	Release of straining	Decrease	No change	Rapid increase	Increase in venous return due to sudden decrease in intrathoracic pressure Recovery of blood volume in the pulmonary vascular bed
IV	Spontaneous breathing after the release of straining	Increase → return to baseline	Decrease → return to baseline	Increase → return to baseline	Increased sympathetic activity and increased venous return. Reactive increase in vagal activity

#### Phase II

In phase II, aortic pressure and pulse pressure gradually decrease due to decreased venous return to the left ventricle (imperceptible pulse) [1]. Sustained straining increases extrathoracic venous pressure and maintains venous return to some extent [5–7]. In phase II, reactive tachycardia occurs due to baroreceptor activation [1]. Although most heart murmurs decrease with reduced stroke volume, murmurs increase in HOCM because of worsening of left ventricular outflow tract obstruction [1]. In mitral valve prolapse, the mitral valve leaflet may move upward, making the clicking phase faster and increasing the murmur [1]. When the murmur increases during the Valsalva maneuver, HOCM or mitral valve prolapse are strongly suggested, although the sensitivity is low [1].

#### Phase III

In phase III, immediately after strain release, a sudden decrease in intrathoracic pressure in addition to the pre-existing decrease in venous return to the left ventricle as well as cardiac output causes decrease in aortic pressure for 1–2 s [1, 4]. Thereafter, venous return rapidly increases in response to decrease in intrathoracic pressure [5–7].

#### Phase IV

In early phase IV, venous return increases two to three folds above baseline, although this is sustained for only one to two heartbeats and then returns to baseline in a few heartbeats [5–7]. Aortic and pulse pressures increase owing to the recovery of left ventricular preload [1]. Systemic blood pressure overshoots compared to baseline. This is attributed to a reactive increase in sympathetic activity caused by a decrease in blood pressure in phase III. At that time, vagal activity increases in response, leading to bradycardia. No overshoot or bradycardia occurs in patients with autonomic dysfunction [1].

### Abnormal response to the Valsalva maneuver

These reactions change under pathological conditions. Because echocardiographic findings are described in detail in each discussion section, only the hemodynamic changes are described here.

At the end of phase II (strain phase), reduced preload leads to a decrease in left ventricular cavity size, thereby exacerbating left ventricular outflow tract obstruction and increasing the heart murmur of HOCM [1].

In phase IV (release phase), although most heart murmurs increase, murmurs in HOCM decrease or even disappear [1]. The murmurs originated from right-sided valves return to the original level in one to two heartbeats, whereas the murmurs from left-sided valves return to the original level after five to six heartbeats. This difference is due to the delayed increase in pulmonary venous return [1].

**Fig. 2:**
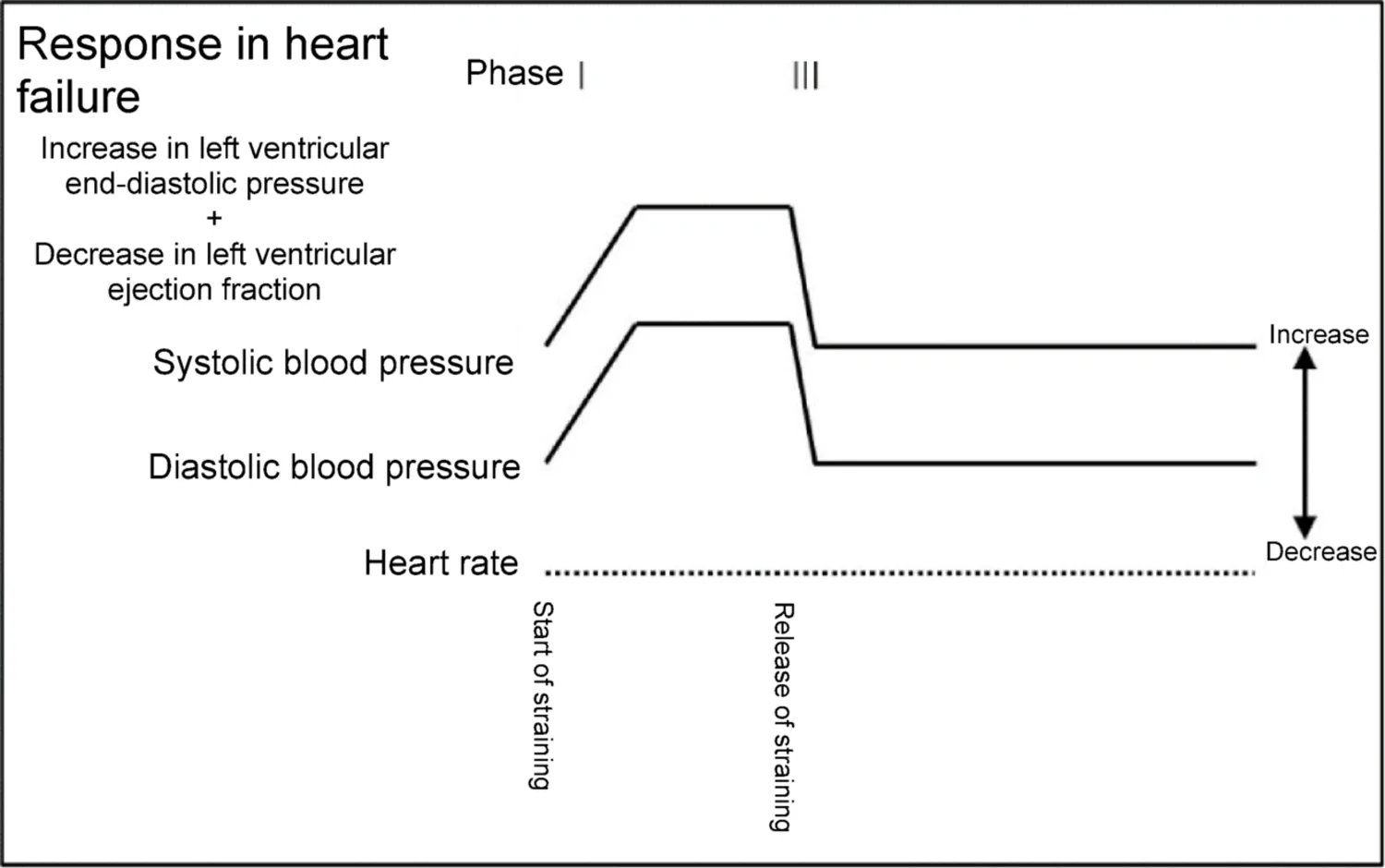
Abnormal response to the Valsalva maneuver in heart failure (square wave response) (prepared based on Reference 1)

In the heart failure state, venous return to the left ventricle barely reduces during the Valsalva maneuver due to pulmonary vascular congestion; the stroke volume in the left ventricle will not decrease. Therefore, a decrease in blood pressure/pulse pressure in phase II or overshoot in phase IV will not occur, and the pressure change will become the square wave (Fig. 2) [1]. This phenomenon is also recognized as a marker of congestion in the Nohria–Stevenson bedside classification of heart failure [8]. Although square-wave response of blood pressure to the Valsalva maneuver is difficult to clearly detect without an arterial catheter in place, it may be inferred from the absence of an imperceptible pulse or of changes in the LV inflow Doppler waveform. An intermediate response was observed in patients with New York Heart Association Functional Classification class II symptoms, mild congestion, or mild cardiac dysfunction.

Increased vagal reflex activity in phase IV has been used to prevent paroxysmal supraventricular tachycardia [1].

Moreover, a normal response to the Valsalva maneuver is less likely to occur in patents with autonomic nervous system imbalance, usage of some types of drugs and decreased intravascular volume owing to bleeding and so on [1].

### Risk of the Valsalva maneuver

Generally, Valsalva maneuver-related complications are considered minor and infrequent [9]. However, problematic complications may occur in patients with underlying diseases. The Valsalva maneuver may cause arrhythmia, chest pain, and syncope (in older patients with reduced autonomic response to decreased venous return). In patients with hypertension, excessive bradycardia may occur in phase I [1]. Coronary blood flow decreases in patients with angina pectoris when using the Valsalva maneuver, which requires caution [1].

## Test implementation

### Confirmation items before test implementation

Careful history taking is advised to confirm whether the patient has contraindicated diseases in Table 2 before performing the test.

**Table 2:** Patients contraindicated for stress echocardiography using the Valsalva maneuver

1.	Acute coronary syndrome within 48 h of onset
2.	Uncontrolled hypertension/heart failure/respiratory failure
3.	Symptomatic severe aortic stenosis
4.	Severe obstructive hypertrophic cardiomyopathy (left ventricular outflow tract pressure gradient > 90 mmHg)
5.	Fatal arrhythmia
6.	Acute phase of acute aortic dissection, impending ruptured aortic aneurysm
7.	Patients who do not cooperate
8.	Patients considered inappropriate by the attending physician

### Pathological conditions requiring caution

Left ventricular outflow tract obstruction with a resting pressure gradient > 50 mmHg, hemoptysis, bloody sputum, pneumothorax, hyperventilation syndrome, and acute inflammatory disease.

### Implementation by sonographers only

Because this test is performed as an extension of routine tests at many institutions, it is often performed only by sonographers. Physicians should readily be reached to manage sudden changes in patients. The Valsalva maneuver should be performed where the patients readily be transferred to a room equipped for oxygen administration, suction, and an emergency cart accessible, in the event of a sudden change.

## Valsalva maneuver for each pathological condition

### Heart failure

#### Purpose

When differentiating a normal left ventricular inflow velocity waveform from a pseudonormal pattern is difficult, the Valsalva maneuver should be performed to facilitate the diagnosis [10, 11]. The Valsalva maneuver may also be performed to evaluate the reversibility of restrictive filling pattern of the left ventricular inflow [10, 11]. These are performed in phase II of the Valsalva maneuver to determine E-wave reduction via decreased left ventricular filling pressure caused by decreased venous return.

#### Inage acquisition during the Valsalva maneuver

**Fig. 3:**
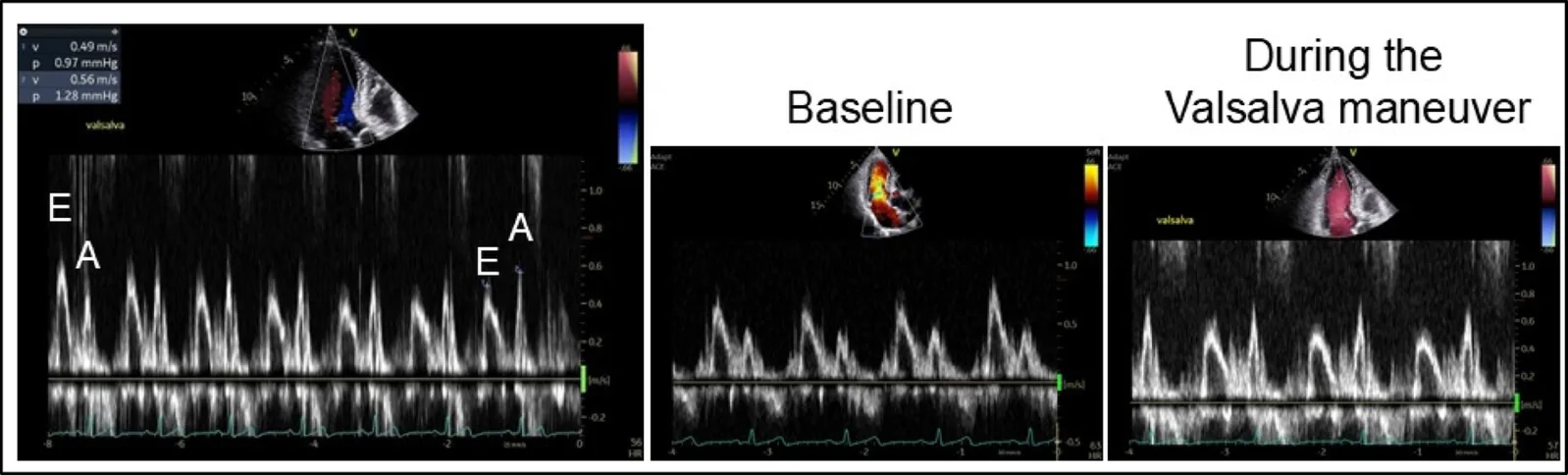
Change in left ventricular inflow velocity waveform during the Valsalva maneuver. Owing to the Valsalva maneuver, the E wave decreased and the A wave increased, with an E/A ratio of < 1. The left ventricular inflow velocity waveform was classified as pseudonormal pattern from these findings

A left ventricular inflow waveform is observed during the Valsalva maneuver. The sweep speed on pulsed-wave Doppler images should be decreased to ≤ 50 mm/s, and the left ventricular inflow waveform should be recorded for 10 to 12 s in phase II of the maneuver where straining was started (Fig. 3) [11]. An annotation to show the application of the Valsalva maneuver is preferred to be indicated in the recorded image.

#### Evaluation items

Changes in the E/A ratio and A-wave peak velocity owing to the Valsalva maneuver.

#### Interpretation of the test

The pseudonormal filling pattern is diagnosed by assessing positivity when the E/A ratio becomes < 1 or the A-wave peak velocity increases during the Valsalva maneuver [11]. In the normal filling pattern, both E and A waves show decreased velocities during the maneuver [10, 12]. In the case of a restrictive filling pattern, if the E/A ratio becomes < 1 during the Valsalva maneuver, it is considered reversible, and if it remains restrictive, it is assessed as an irreversible restrictive pattern [10].

#### Impact on treatment

If a pseudonormal or irreversible restrictive filling pattern is determined by the Valsalva maneuver, increased left ventricular filling pressure is suspected. The attending physician will evaluate the results and prevent the exacerbation of heart failure by optimizing volume status.

### HOCM, left ventricular outflow tract obstruction

#### Purpose

In patients with hypertrophic cardiomyopathy or sigmoid interventricular septum, the Valsalva maneuver is performed to induce left ventricular outflow tract obstruction when (1) the patient complains shortness of breath, but no significant left ventricular outflow tract obstruction or (2) systolic anterior motion (SAM) of the mitral valve is observed whereas no significant pressure gradient is detected in the left ventricular outflow tract [13]. Since left ventricular outflow tract obstruction is enhanced by reduced preload, [14] the decrease in venous return in phase II of the Valsalva maneuver induces or further enhance the obstruction.

#### Image acquisition during the Valsalva maneuver

First, the left ventricular outflow tract is monitored in the apical long-axis view or apical five-chamber view during the Valsalva maneuver. Changes in SAM on two-dimensional images and mosaic blood flow in the left ventricular outflow tract and mitral regurgitation associated with SAM on color Doppler images are also observed. Continuous-wave Doppler images of the left ventricular outflow tract are then recorded during the same maneuver. In patients with concomitant mitral regurgitation due to SAM, continuous wave Doppler imaging at the site of left ventricular outflow tract obstruction should be securely recorded to avoid mitral regurgitation [15]. If it is difficult to distinguish whether the blood flow velocity is that of the left ventricular outflow tract or of mitral regurgitation, continuous wave Doppler images should be recorded after ensuring that the beam is directed to the left ventricular ejection flow on color Doppler images during the Valsalva maneuver. Continuous wave Doppler waveforms of mitral regurgitation are faster and longer in duration than left ventricular outflow tract obstruction and are characterized by a peak at mid-systole, which is used for differentiating between mitral regurgitation and left ventricular outflow tract obstruction [15]. In addition, when mitral regurgitation is directed posteriorly, the left ventricular ejection flow moving toward the probe can be separated from the continuous wave Doppler waveform of mitral regurgitation that is moving away, as observed from the parasternal long-axis view.

#### Evaluation items

**Fig. 4:**
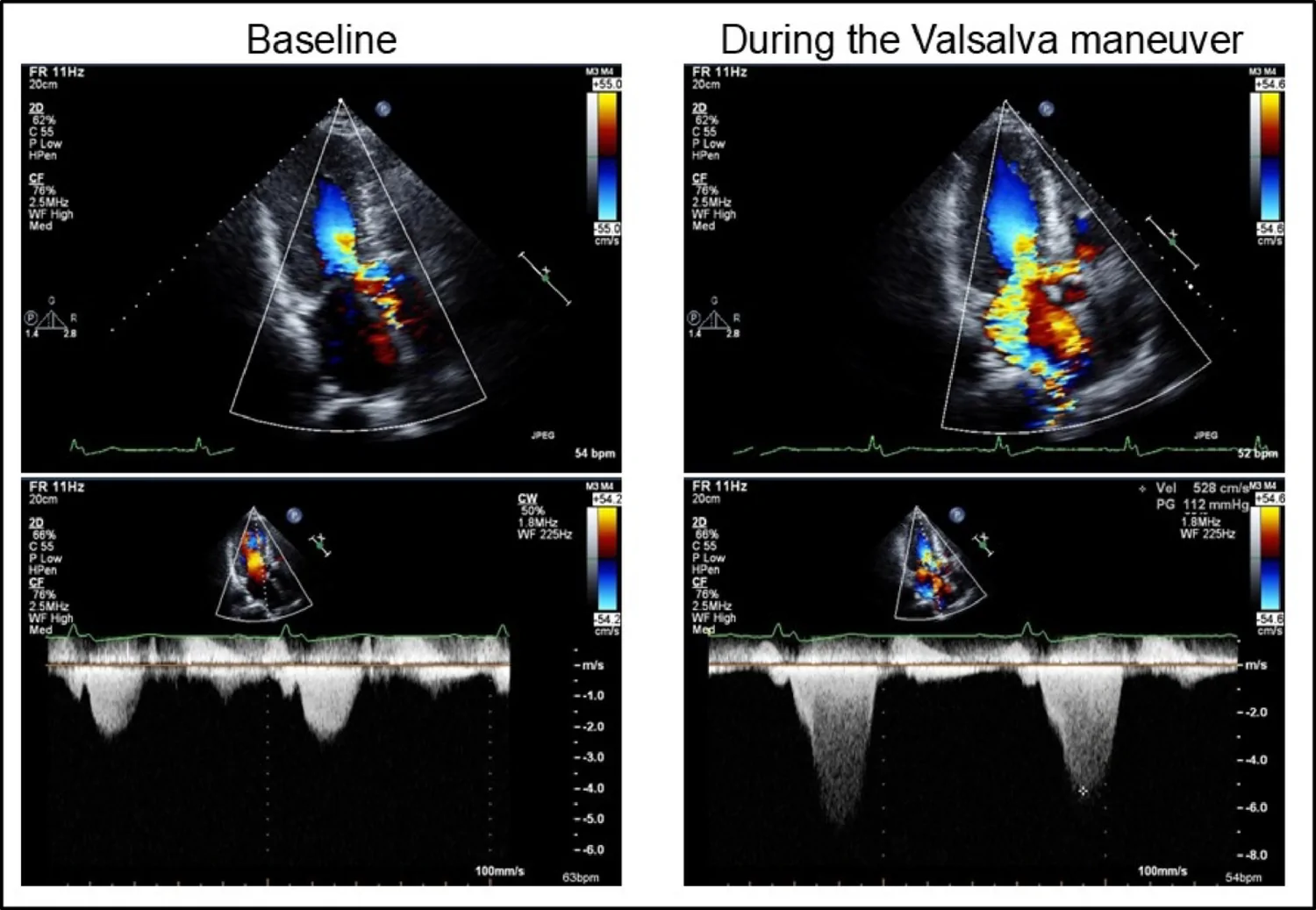
Valsalva maneuver for latent left ventricular outflow tract obstruction. Apical long-axis color Doppler image (top) and a continuous-wave Doppler image of the left ventricular outflow tract (bottom). The Valsalva maneuver causes mitral regurgitation associated with the systolic anterior motion of the mitral valve, increasing the mosaic flow in the left ventricular outflow tract. Continuous-wave Doppler imaging, which avoids mitral regurgitation, showed a pressure gradient of 112 mmHg, indicating outflow tract obstruction

The presence or absence of SAM appearance or enhancement during the Valsalva maneuver, appearance of mosaic blood flow in the left ventricular outflow tract, and the maximum pressure gradient of the left ventricular ejection flow (Fig. 4).

#### Interpretation of the test

A peak left ventricular outflow tract pressure gradient ≥ 30 mmHg during Valsalva maneuver is considered positive and indicates latent left ventricular outflow tract obstruction [13, 14]. A left ventricular outflow tract pressure gradient of 50 mmHg is generally used as the threshold for therapeutic intervention in patients with HOCM [13, 14].

#### Description of test results

If the result was positive, the examiner reports that a significant left ventricular outflow gradient was induced using the Valsalva maneuver. If it was negative, it is reported that an outflow obstruction was not induced while induction was performed. Since multiple factors are associated with the enhancement of left ventricular outflow tract obstruction, some outflow tract obstructions are not induced by the Valsalva maneuver. Therefore, in patients in whom left ventricular outflow tract obstruction is strongly suspected clinically but significant outflow tract obstruction is not induced, other stress methods such as exercise stress, standing stress, and sitting stress should be considered.

### Patent foramen ovale (*PFO*)

#### Purpose

Echocardiography during the Valsalva maneuver with saline contrast should be performed in patients with embolic stroke of undetermined source with suspected paradoxical embolism involving PFO to induce a right-to-left shunt via PFO [16–18]. A right-to-left atrial shunt is induced by transiently increasing the right atrial pressure through increased venous return during phase III of the Valsalva maneuver.

#### Image acquisition during the Valsalva maneuver

The contrast agent is prepared by mixing 1 mL of air and 8–9 mL of normal saline [16, 19]. The method of mixing 1 mL of blood aspirated from a patient, 1 mL of air, and 8 mL of normal saline reportedly produces better contrast effects than the method using air alone [20] and has been widely used. If blood aspiration is technically impossible, one drop of diazepam may be added instead of blood. However, this is an off-label use. Although no such cases have been reported to date, there is concern that the use of blood may be associated with embolism due to the formation of small thrombi. However, to prevent air contamination due to insufficient agitation, using > 1 mL of air should be avoided, and care should be taken to avoid injecting coagulated blood outside the body for prolonged periods.

A cut plane that allows simultaneous visualization of all four cardiac chambers, such as apical four-chamber or parasternal four-chamber view, should be obtained, and the patient should then be instructed to perform a forceful straining maneuver [21]. At that time, an imaging plane should be selected in which the right ventricle is placed as little as possible in front of the left ventricle, so as to minimize artifacts caused by bubbles. The strength of stress can be maintained if it is applied while compressing the abdomen. Therefore, abdominal compression should be performed as concomitantly as possible. Agitated saline is rapidly administered intravenously at the onset of straining and release of straining when the entire right heart is imaged, and the appearance or absence of contrast in the left heart system is assessed.

#### Evaluation items

A positive result is defined as contrast appearing in the left heart within 3 heartbeats after release of the Valsalva maneuver, with grading of severity.

#### Interpretation of the test

**Fig. 5:**
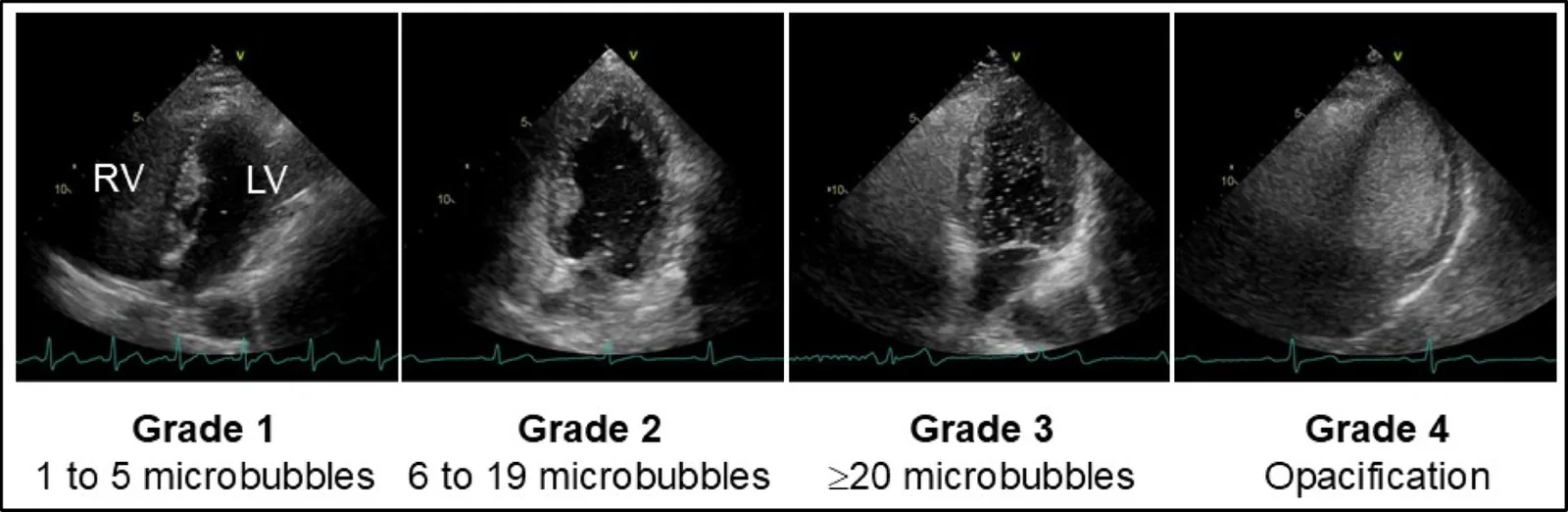
Grading of the right to left shunt in **microbubble test**. LV, left ventricle; RV, right ventricle

Images are considered negative when no contrast is observed in the left heart. When contrast appears in the left heart, it is classified into four grades according to the degree, although the cutoff value varies among reports [16–18]. The cutoff values described in the guidelines of the Japan Stroke Society are introduced in this guidance (Fig. 5).

#### Description of results

The presence or absence of contrast in the left heart system and its degree is described in the report. If the patient has an intrapulmonary shunt, contrast appears in the left heart without Valsalva maneuver a few seconds after the appearance of contrast in the right heart. Therefore, the results of the observations during normal respiration might also be described.

All authors are members of the Echocardiogram Guideline Committee of the Japanese Society of Echocardiography in 2026.

## Appendix

### Editorial supervision

Echocardiogram Guideline Preparation Committee: Chairperson: Hiroyuki Okura, Department of Cardiovascular Medicine, Gifu University Graduate School of Medicine. Member: Masashi Amano, Department of Heart Failure/Transplantation, National Cerebral and Cardiovascular Center. Member: Hiromi Umeda, Kokura Kinen Hospital. Member: Masaru Obokata, Department of Cardiovascular Medicine, Gunma University Graduate School of Medicine. Member: Nobuyuki Kagiyama, Department of Remote Cardiovascular Management, Juntendo University School of Medicine. Member: Masao Daimon, Department of Cardiology, Tokyo Women’s Medical University. Member: Hidekazu Tanaka, Division of Cardiology, Department of Medicine, Kindai University Faculty of Medicine. Member: Norihisa Toh, Department of Cardiovascular Medicine, Okayama University School of Medicine.

External Evaluators: Yukio Abe, Department of Cardiovascular Medicine, Osaka City General Hospital. Kumiko Hirata, Department of Cardiovascular Medicine, Kishiwada Tokushukai Hospital.
